# Pharmacogenomic biomarker information differences between drug labels in the United States and Hungary: implementation from medical practitioner view

**DOI:** 10.1038/s41397-019-0123-z

**Published:** 2019-12-02

**Authors:** Reka Varnai, Istvan Szabo, Greta Tarlos, Laszlo Jozsef Szentpeteri, Attila Sik, Sandor Balogh, Csilla Sipeky

**Affiliations:** 1 Department of Primary Health Care, Medical School, University of Pécs, H-7623 Pécs, Rákóczi u 2, Hungary; 2 Doctoral School of Health Sciences, Faculty of Health Sciences, University of Pécs, H-7621 Pécs, Vörösmarty u 4, Hungary; 3 Institute of Sport Sciences and Physical Education, University of Pécs, H-7624 Pécs, Ifjúság útja 6, Hungary; 4 Faculty of Sciences, Doctoral School of Biology and Sportbiology, University of Pécs, H-7624 Pécs, Ifjúság útja 6, Hungary; 5 Faculty of Pharmacy, University of Pécs, H-7624 Pécs, Rokus u 2, Hungary; 6 Institute of Transdisciplinary Discoveries, Medical School, University of Pécs, H-7624 Pécs, Szigeti út 12, Hungary; 7 Insitute of Biomedicine, University of Turku, Kiinamyllynkatu 10, FI-20520 Turku, Finland

**Keywords:** Biomarkers, Genomics

## Abstract

Pharmacogenomic biomarker availability of Hungarian Summaries of Product Characteristics (SmPC) was assembled and compared with the information in US Food and Drug Administration (FDA) drug labels of the same active substance (July 2019). The level of action of these biomarkers was assessed from The Pharmacogenomics Knowledgebase database. From the identified 264 FDA approved drugs with pharmacogenomic biomarkers in drug label, 195 are available in Hungary. From them, 165 drugs include pharmacogenomic data disposing 222 biomarkers. Most of them are metabolizing enzymes (46%) and pharmacological targets (41%). The most frequent therapeutic area is oncology (37%), followed by infectious diseases (12%) and psychiatry (9%) (*p* < 0.00001). Most common biomarkers in Hungarian SmPCs are *CYP2D6, CYP2C19*, estrogen and progesterone hormone receptor (ESR, PGS). Importantly, US labels present more specific pharmacogenomic subheadings, the level of action has a different prominence, and offer more applicable dose modifications than Hungarians (5% vs 3%). However, Hungarian SmPCs are at 9 oncology drugs stricter than FDA, testing is obligatory before treatment. Out of the biomarkers available in US drug labels, 62 are missing completely from Hungarian SmPCs (*p* < 0.00001). Most of these belong to oncology (42%) and in case of 11% of missing biomarkers testing is required before treatment. In conclusion, more factual, clear, clinically relevant pharmacogenomic information in Hungarian SmPCs would reinforce implementation of pharmacogenetics. Underpinning future perspective is to support regulatory stakeholders to enhance inclusion of pharmacogenomic biomarkers into Hungarian drug labels and consequently enhance personalized medicine in Hungary.

## Introduction

Pharmacogenomics (PGx) is one of the precision medicine (PM) tools to be applied to maximize treatment effectiveness, while limit the drug toxicity by differentiating responders from nonresponders to medications, based on an individual’s genetic constitution [1]. Pharmacogenomic information may be provided in drug labeling to inform healthcare providers about the impact of genotype on response to a drug through description of relevant genomic markers, functional effects of genomic variants, dosing recommendations based on genotype, and other applicable genomic information [2]. This can describe variability in clinical response and drug exposure, risk of adverse events, genotype-specific dosing, mechanisms of drug action, polymorphic drug target and disposition genes or trial design features [3].

Information on PGx biomarkers and laboratory testing provides the resource for practicing medical doctors to apply personalized medicine in clinic [4]. In order to implement PGx in clinical setting, practicing doctors need to have both information on PGx biomarkers or guidelines implementing the use of biomarkers, and available laboratory tests as input, and handy implementation tools to be able to generate output in clinics.

The drug labeling for some, but not all, of the products includes specific actions to be taken based on the PGx biomarker information. This information can appear in different sections of the labeling depending on the actions [3].

One would expect regulations for drugs and diagnostics not to differ significantly between countries, given that regulatory authorities evaluate the same scientific data generated in an increasingly globally harmonized context [5]. Despite international regulatory harmonization, implementation of the pharmacogenomic information in official drug labeling shows wide range of geographical variety [6]. The US Food and Drug Administration (FDA) and the European Medicines Agency (EMA) work jointly and in multiple ways on scientific evaluation of drugs to ensure that pharmacogenomic strategies are applied appropriately in all phases of drug development. EMA is responsible for the centralized marketing authorization applications in the European Union and some additional countries. Once granted by the European Commission, the centralized marketing authorization is valid in all European Union Member States, in Hungary as well. However, several drugs have undergone the Hungarian national marketing authorization process previously, therefore the PGx information might be not updated.

The ultimate aim and rationale of this study is to:

1. Provide an evaluation of current status of PGx biomarker information present in Hungarian drug labels.
2. Summarize the potential needs of medical practitioners, healthcare providers.
3. Identify the gaps of PGx implementation and potential solutions.

## Materials and methods

All data presented in this work have been collected in July 2019. Consequently, the US FDA information on available pharmacogenomic biomarkers in drug labeling represents the most up-to-date current content as of 26 March 2019 (https://www.fda.gov). The Hungarian Summaries of Product Characteristics (SmPCs) of the same active substance were assessed from the National Institute of Pharmacy and Nutrition database of Hungary (www.ogyei.gov.hu/gyogyszeradatbazis/). PGx information on the level of action was collected on PharmGKb^®^ (www.pharmgkb.org) and compared with the same information from the Hungarian SmPCs. Identical data collection was performed in 2017 spring, providing the opportunity to have an overview about the dynamic change of the implementation of PGx information in Hungarian drug labels.

Biomarkers in our investigation include but are not limited to germline or somatic gene variants (polymorphisms, mutations), functional deficiencies with a genetic etiology, gene expression differences, and chromosomal abnormalities; specific protein biomarkers that are used to select treatments for patients are also included.

The investigation does not include nonhuman genetic biomarkers (e.g., microbial variants that influence sensitivity to antibiotics), biomarkers that are used solely for diagnostic purposes (e.g., for genetic diseases) unless they are linked to drug activity or used to identify a specific subset of patients in whom prescribing information differs, or biomarkers that are related to a drug other than the referenced drug (e.g., influences the effect of the referenced drug as a perpetrator of an interaction with another drug).

For drugs that are available in multiple dosage forms, salts, or combinations, a single-representative product is listed. In the case of combination products, the single agent associated with the biomarker is listed unless the agent is only approved as a combination product, in which case all agents are listed.

We assessed PGx level of action categories according to PharmGKb^®^ [7] of the doctor targeted section of Hungarian drug label as (1) testing required, (2) testing recommended, (3) actionable with dosing info, (4) actionable, and (5) informative.

In order to measure the statistical differences, two-sided *p* values were calculated using Pearson’s chi-squared test or Fisher’s exact test. A *p* value < 0.05 was considered to indicate a statistically significant result. Statistical analyses were performed applying Microsoft^®^ Excel^®^ for Mac^®^ 2011 and IBM^®^ SPSS^®^ Statistics Version25 for Mac (SPSS Inc., Chicago, IL, USA).

## Results

**Fig. 1:**
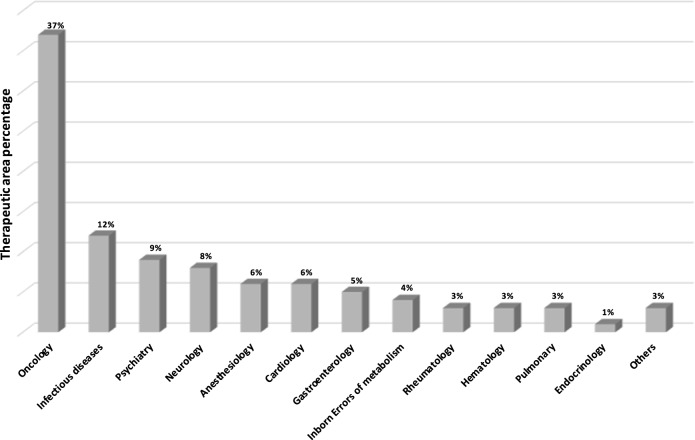
Therapeutic areas of drugs with pharmacogenomic information in their labeling in Hungary

We identified 264 drugs in the US FDA Table of Pharmacogenomic Biomarkers in Drug Labeling after excluding duplicate active ingredients. Out of these 264 active ingredients we were able to identify 195 (74%) through the website of the National Institute of Pharmacy and Nutrition in Hungary being available in Hungary (Table 1). Among the 195 drugs, 145 (75%) have PGx information included in the Hungarian product summary. Important to note that while taking a point-in-time snapshot, the number of drugs with PGx information in the drug label has elevated in the US with 57% vs in Hungary with 46% in last 26 months. PGx information is partially present in drug label of 20 (10%), completely missing from drug label of 30 (15%) available active ingredients in Hungary compared with US FDA (Table 1, italic and bold, respectively). These drugs without PGx biomarker information in their label belong to diverse therapeutic areas (23% oncology, 23% anesthesiology, 20% infectious diseases, 7% cardiology, 7% inborn error, 7% rheumatology, 3% dermatology, 3% hematology, 3% psychiatry, and 3% pulmonology). The 69 drugs not available in Hungary are listed in Supplementary Table 1. The distribution of therapeutic areas of drugs with PGx information in their labeling is presented on Fig. 1. The most frequent therapeutic area is oncology (37%), followed by infectious diseases (12%), psychiatry (9%), and neurology (8%) (*χ*^2^ *p* < 0.00001).

**Table 1:** Drugs in the Hungarian National Institute of Pharmacy and Nutrition database with complete (*n* = 145), with partial (*n* = 20 italic), and without (*n* = 30 bold) pharmacogenomic information in their Summary of Product Characteristics^a^

Abacavir	Diazepam	Lenalidomide	Ponatinib
Abemaciclib	Dinutuximab	Lesinurad	Prasugrel
Afatinib	Docetaxel	Letrozole	Pyrazinamide
Alectinib	Dolutegravir	**Lidocaine**	Prilocain
Amifampridine	Donepezil	*Lorlatinib*	Propafenone
Amitriptyline	Drospirenone	Lumacaftor	Propranolol
Anastrozole	Duloxetine	**Lusutrombopag**	Quinidine
Aripiprazole	Durvalumab	**Mepivacaine**	Quinine Sulfate
Arsenic Trioxide	Efavirenz	*Mercaptopurine*	Rabeprazole
**Articaine**	Elbasvir	Methylene Blue	**Raloxifene**
Atomoxetine	Eliglustat	*Metoclopramide*	Raltegravir
Avatrombopag	Elosulfase	Metoprolol	*Rasburicase*
Avelumab	Eltrombopag	*Midostaurin*	Ribociclib
Azathioprine	Encorafenib	Migalastat	**Rifampin**
Binimetinib	*Eribulin*	Mirabegron	Risperidone
Blinatumomab	Erlotinib	Mivacurium	Rituximab
Bosutinib	Escitalopram	Mycophenolic Acid	**Rivaroxaban**
*Brentuximab*	Esomeprazole	Nebivolol	**Ropivacaine**
Vedotin	Ethinyl Estradiol	Neratinib	Rosuvastatin
Brexpiprazole	Everolimus	Nilotinib	Rucaparib
Brigatinib	*Exemestane*	Niraparib	**Sevoflurane**
Brivaracetam	Fesoterodine	Nitrofurantoin	**Sodium**
Busulfan	Fluorouracil	*Nivolumab*	**Phenylbutyrate**
Cabozantinib	Fluoxetine	Nusinersen	**Sofosbuvir**
Capecitabine	**Flurbiprofen**	Obinutuzumab	Sulfadiazine
Carbamazepine	**Flutamide**	Olaparib	*Sulfamethoxazole*
Carglumic Acid	Fluvoxamine	Olaratumab	*Sulfasalazine*
Cariprazine	**Formoterol**	*Ombitasvir*	Talazoparib
Carvedilol	Fulvestrant	Paritaprev	**Tamoxifen**
**Ceftriaxone**	Galantamine	**Ombitasvir**	Tamsulosin
Celecoxib	Gefitinib	Omeprazole	Tetrabenazine
Ceritinib	Glimepiride	Ondansetron	**Tetracain**
Cerliponase Alfa	**Goserelin**	Osimertinib	Tezacaftor
Cetuximab	Grazoprevir	Ospemifene	Ticagrelor
Chloroquine	Ibrutinib	Oxcarbazepine	Toremifene
**Cisplatin**	Imatinib	**Oxymetazoline**	Tramadol
Citalopram	**Imipramine**	Palbociclib	*Trametinib*
Clobazam	Indacaterol	Palonosetron	Trastuzumab
Clomipramine	Inotersen	*Panitumumab*	**Tretinoin**
Clopidogrel	Inotuzumab	Pantoprazole	*Trimethoprim*
Clozapine	Ozogamicin	**Parathyroid**	Umeclidinium
Cobimetinib	*Ipilimumab*	**Hormone**	**Ustekinumab**
Codeine	Irinotecan	Paroxetine	Valproic Acid
Crizotinib	Isoflurane	Patisiran	Vemurafenib
*Dabrafenib*	Isoniazid	Pazopanib	*Venetoclax*
Daclatasvir	**Isosorbide**	**Peginterferon**	Velpatasvir
Dacomitinib	**Mononitrate**	**Alfa**-**2b**	Venlafaxine
Darifenacin	Ivacaftor	*Pembrolizumab*	**Vincristine**
*Dasabuvir*	Lacosamide	Pertuzumab	Voriconazole
Dasatinib	Lansoprazole	Phenytoin	Vortioxetine
Dexlansoprazole	Lapatinib	**Piroxicam**	Voxilaprevir
Dextromethorphan	**Ledipasvir**		Warfarin

The table represents the status of 2019 July

^a^Out of 264 FDA listed drugs with pharmacogenomic biomarkers in drug labeling, 195 are marketed in Hungary

As one drug’s PGx can be affected by more than one specific biomarker, the identified 165 drugs with PGx data (including drugs with partially present data) dispose 222 biomarkers in the Hungarian SmPCs summarized in Table 2. In the Hungarian SmPCs, we identified information either on metabolizing enzymes (*n* = 102, 46%), pharmacological targets (*n* = 90, 41%), or other features (*n* = 30, 13%).

**Table 2:** Pharmacogenomic biomarkers in Hungarian Summaries of Product Characteristics of 165 drugs

Biomarker		Frequency (*n* = 222)	Percentage (%)
Metabolizing enzyme (*n* = 102)	CYP2D6	40	18.00
	CYP2C19	18	8.01
	G6PD	10	4.05
	UGT1A1	7	3.02
	CYP2C9	6	2.07
	CYP2B6	3	1.04
	DPYD	3	1.04
	NAT1	2	0.09
	TPMT	2	0.09
	BCHE	1	0.05
	CYP1A2	1	0.05
	CYP3A5	1	0.05
	GALNS	1	0.05
	GLA	1	0.05
	HPRT1	1	0.05
	NAGS	1	0.05
	NAT2	1	0.05
	SLCO1B1	1	0.05
	Urea cycle disorder	1	0.05
	VKORC1	1	0.05
Target (*n* = 90)	ESR, PGR	15	6.07
	ERBB2	12	5.05
	BCR-ABL1	8	3.06
	BRAF	8	3.06
	EGFR	6	2.07
	ALK	5	2.03
	Del 5q/17p/11q	5	2.03
	RAS	5	2.03
	BRCA	4	1.80
	CD274	4	1.80
	CFTR	2	0.09
	KIT	2	0.09
	MS4A1	2	0.09
	TTR	2	0.05
	FIP1L1-P	1	0.05
	FLT3	1	0.05
	PDGFRA	1	0.05
	PDGFRB	1	0.05
	PML-RARA	1	0.05
	RET	1	0.05
	ROS1	1	0.05
	SMN2	1	0.05
	TNFRSF8	1	0.05
	TP53	1	0.05
Other (*n* = 30)	HLA-B	5	2.03
	IFNL3	5	2.03
	F5	2	0.09
	HLA-A	2	0.09
	PROC	2	0.09
	PROS1	2	0.09
	SERPINC1	2	0.09
	Nonspecific (congenital methemoglobinemia)	1	0.05
	CYB5R	1	0.05
	F2	1	0.05
	HLA-DQA1	1	0.05
	IGH	1	0.05
	MYCN	1	0.05
	NUDT15	1	0.05
	POLG	1	0.05
	RYR1	1	0.05
	TPP1	1	0.05

The table represents the status of 2019 July

The most common biomarkers in Hungarian SmPCs are the *CYP2D6* (*n* = 40, 18%), the *CYP2C19* (*n* = 18, 8%), the estrogen and progesterone hormone receptors (ESR, PGR, *n* = 15, 6%), the *ERBB2* (*n* = 12, 5%), and the *G6PD* (*n* = 10, 4%). We also observed that none of the SmPCs containing PGx biomarker data has any PGx evidence specifically for Hungarian population, neither on clinical endpoints nor on pharmacokinetics.

Pharmacogenomic biomarkers influence the drug treatment on several different ways, thus one biomarker can have more than one impact. According to the Hungarian product summary, the aim of pharmacogenomic biomarker use can be the following: effects efficacy (*n* = 84), indicates toxicity (*n* = 67), belongs to the inclusion criteria (*n* = 67), belongs to the exclusion criteria (*n* = 24) because of elevated toxicity risk or effect dosage (*n* = 18). Moreover, 53 biomarkers (24% of all) are involved in drug–drug interaction management as dose modification or elevated toxicity risk is connected to the presence of enzyme inhibitor/inductor irrespective of the pharmacogenomic background. Highly importantly, eight biomarkers (4 %) are factual in point of dosing and formulate exact algorithm to manage gene–drug interaction.

Out of the biomarkers available in US drug labels, 62 (22%) are missing from the Hungarian SmPCs (*p* < 0.00001, Fisher’s exact test). Our dynamic update shows that the percentage of missing PGx data in Hungarian drug labels has doubled in last 26 months as a result of accelerated PGx biomarker implementation in US FDA drug labeling. Most of the missing pharmacogenomic biomarkers belong to the therapeutic area of oncology (42%), followed by anesthesiology (18%), infectious diseases (13%); hematology (8%); cardiology, dermatology, gastroenterology, inborn errors of metabolism, psychiatry, pulmonology, rheumatology represent minor proportions (<4% each).

In order to be able to compare the level of action of PGx biomarkers between Hungary and the United States, we extracted the information from the Hungarian SmPCs for US FDA approved drugs available in Hungary and compared with the level of action available on The Pharmacogenomics Knowledgebase (www.pharmgkb.org) (Table 3). Testing is required at 72 biomarkers (25 %) in Hungary, from which 66 (92%) belong to field of oncology. In United States, in case of 79 (28%) biomarkers is testing obligatory before treatment. Four (1%) biomarkers in Hungarian drug labels are ranked into testing recommended category, six (2%) biomarkers in the United States. PGx information is actionable at 95 (34%) biomarkers in Hungary, compared with 108 (38%) in the United States. Out of the actionable biomarkers, 14 (5%) biomarkers dispose exact dosing adjustment in PharmGKB recommendation, but only eight (3%) of them are ranked into the same category in Hungary. The six (3%) remaining biomarkers predispose only actionable PGx data without dosing info in Hungarian drug inserts. Fifty-one (18%) biomarkers have informative PGx data in Hungarian drug label; however, in the United States 77 (27%) biomarkers are counted into this category (*p* = 0.009). Even from FDA US biomarkers 14 (5%) are missing from PharmGKB, which shows generally a rather delayed implementation of PGx information. This is the case for 62 (22%) biomarkers for Hungarian SmPC’s (*p* < 0.00001).

**Table 3:** Comparison of the level of action of pharmacogenomic information acquired from Hungarian SmPCs and the PharmGKB annotation of US FDA pharmacogenomic biomarkers (*n* = 284)

Pharmacogenomic level of action	Hungarian SmPC, *n* (%)	US FDA on PharmGKB, *n* (%)	*p* value*
Testing required	72 (25)	79 (28)	0.506
Testing recommended	4 (1)	6 (2)	0.523
Actionable	95 (34)	108 (38)	0.255
Informative	51 (18)	77 (27)	**0.009**
Missing	62 (22)	14 (5)	**<0.00001**

Based on 2019 July status

*χ^2^ test; statistically significant difference is marked with bold, p < 0.05;

Talking about the PGx level of action, out of the 62 missing biomarkers from Hungarian SmPC’s 7 (11%) belong to testing required category, 27 (44%) belong to actionable PGx category and 21 (29%) belong to informative PGx category according to PharmGKB.

In order to implement PGx in everyday medical practice, we need to translate PGx biomarker information into drug level. It practically means that partially missing biomarkers in Hungarian SmPCs belong to 20, completely missing biomarkers to 30 drugs shown in Table 1. Notably, after checking the level of action, in case of 7 from these 50 drugs biomarker testing is required before treatment according to PharmGKB. It is of utmost importance that six from these seven drugs belong to oncology medication and therefore define cancer treatment. On the other hand, in case of nine oncology drugs, the Hungarian SmPCs are even stricter than the FDA recommendation and genetic testing is required before treatment.

Hungarian SmPCs mention information on lab test availability at 76 biomarkers (34%). However, the product summary does not ever refer on an exact laboratory in Hungarian drug label. The information on lab test availability is based on clinics internal regulation and doctor’s daily routine either on commercial test or on academic setting.

## Discussion

PM strategies and PGx are becoming more prevalent in research and clinical practice and are integral part of drug development. Therefore, including appropriate pharmacogenomic information and accurate description in drug labels intend to support medical professionals and patients is critical [2, 8].

Territorial differences in drug label content of PGx biomarker information depending on responsible approval agencies do exist. For example, it is well known that cytochrome P450 pharmacogenetic information included in US FDA drug labels present significantly more specific pharmacogenetic information than analogous EU SmPCs [9].

Therefore, comparing labeling of medicines in Hungary versus the United States may identify gaps to solve. While investigating similarities and differences of PGx information in the United States and Hungarian drug label content, we identified that US labels presented significantly more specific pharmacogenetic subheadings than analogous Hungarian SmPCs. As 62 PGx biomarkers are missing completely from Hungarian SmPCs, Hungarian drug labels may need to be supplemented in future with the pharmacogenetic biomarker information in case of these active substances.

Our study demonstrates that the most frequent therapeutic area with pharmacogenomic information in the drug label is oncology both in the United States and in Hungary. This is in line with the EMA statement that PGx information are preferentially present in drug labels having antineoplastic properties [10]. In the field of oncology, pharmacogenetic biomarkers represent a complex combination of germline and somatic variants [11]. Importantly, somatic mutations in tumor cell are increasingly implicated biomarkers in targeted therapy, applied in treatment selection, and are also often associated with treatment efficacy [12]. This is well represented in Hungarian drug labels since the main aim of pharmacogenomic biomarker use is to tailor treatment efficacy. On the other hand, hereditary variants affect pharmacokinetics and pharmacodynamics, and are more often considered to address adverse drug reactions. Tumor sequencing for somatic mutation detection is applied in Hungarian institutions, and produces matched germline information. However, targeted tumor genome sequencing, to provide precision treatment decisions for patients, more relevantly reflects the local practices. Most commonly tested biomarkers in oncology in Hungary are pharmacological targets, where molecular diagnostics is required for patient selection and personalized genotype-directed therapy. For example, EGFR/KRAS/ALK in non-small cell lung carcinoma, or BRAF, NRAS in melanoma, in agreement with the ESMO guidelines [13, 14]. In addition, BRCA1/2 are tested in breast and ovarian cancers, but it is not obligatory. In other tumors there is less consensus.

According to our results, US labels scored the level of action of PGx information on the same overall quality than the analogous Hungarian SmPCs, but the prominence is different. Hungarian SmPCs are stricter regarding oncological drugs than US labels. Rigor towards genetic testing before oncology drug treatment in Hungary may be caused by the high cost of these target molecules, therefore confirmation of efficacy is rather obligatory before treatment. However, the proportion of requirement or recommendation for PGx testing is higher in oncology than in other therapeutic areas in the United States [15]. Of note, FDA offers more applicable information about dose modifications than Hungarian SmPCs. FDA has recognized genetic differences in drug metabolism where clinically relevant drug–drug interactions or gene–drug interactions trigger dose adjustment or use of alternative drugs [16].

Considering differences in gene expression and physiological maturation between pediatric and adult populations, extrapolation of adult pharmacogenetic information in FDA approved pediatric drug labels is not always appropriate [17, 18]. Ontogeny-associated treatment response differences are specifically important in pediatric oncology drugs [18]. Nonetheless, pharmacogenomic biomarker information is commonly based on adult studies both in Hungarian SmPCs and FDA drug labels.

Classification of PGx biomarkers (e.g. metabolizing enzymes, pharmacological targets, and others) is not available in Hungarian data resources. Categorization of biomarkers need to be implemented in Hungarian SmPC’s, in order to clarify PGx information and consequently enhance genetic biomarker testing in daily medical routine.

Pharmacogenetics-related drug-labeling updates do not always result in uniform clinical uptake of pharmacogenetic testing. Lack of simultaneous implementation of newly approved drugs linked to companion diagnostic biomarkers into the clinical practice has several reasons. Potential factors leading to heterogeneity in clinical uptake of pharmacogenetic testing include the strength of supportive evidence (1), which may originate from low contribution of known genetic variant to outcome or incomplete understanding of genetic variation effect; the consequences of a targeted adverse event or treatment failure (2); the availability of alternative agents or dosing strategies (3); the predictive utility of testing (4); test cost-effectiveness, accessibility, and turnaround time (5); reimbursement issues (6); professional society positions (7); or simple general resistance to use of genetic tests (8) [19, 20]. For example, information on lab test availability is unattached to Hungarian drug label and must have different source in the everyday medical work. The crucial solution can be establishment of the Europe-wide database for PGx laboratory test availability. Tough, a limited set of PGx biomarker test is available in Hungary, provided by three university laboratories (Pécs, Budapest, and Debrecen). All available obligatory tests are reimbursed by the Hungarian State Insurance if the genotyping has been done in noncommercial laboratory. The genotyping approach, the laboratory contacted depend on personal practice of the specific doctors. Also, implementation platforms delivering ready-to-apply genetic results in clinic are missing. In order to take advantage of PGx biomarkers in clinical practice integration with other personalized medicine approaches is also needed. On the other hand, preemptive pharmacogenomic testing of actionable genetic markers predicting systemic exposure can be the most future oriented approach to use PGx biomarkers in practice. All of these will unequivocally enhance the rate of uptake of PGx information by medical practitioners.

Acceleration is seen in implementation of PGx info both in the United States and Hungary, though the regulatory dynamics is different. In case regulatory agencies enhance the inclusion of PGx biomarker information in Hungarian drug labels less technical barriers hinder the implementation of PM. The laboratory and professional requirements for all FDA biomarker testing are certainly available in Hungary. Although, pharmacogenomic knowledge of healthcare professionals and the corresponding medical education in PGx [21], as one of the key factors in implementation, need to be improved as well [22].

Hungarian drug labels do not contain any PGx evidence for Hungarian population neither on clinical endpoints nor on pharmacokinetics. Regulatory approval and submission of new drug application are based on international clinical trial’s outcome in Hungary. However, this can be due to the low number of inhabitants in Hungary (ten Million) and the population’s genetic heterogeneity. More focus may be given to the investigation of dose and regimens for special populations before applying for marketing authorization. Consequently, regulators could review dose–exposure–response data with more certainty and better define dose recommendations in the label [23]. For unlicensed drugs we suggest representing PGx information in the SmPCs before marketing authorization such as for drugs under renewal or variation process.

Limitations of the study include the followings. The field of PGx is rapidly advancing, therefore drug labeling is not static. Updating PGx information is a dynamic process and new markers are constantly being added. This is shown by 57% elevation of FDA drugs with PGx biomarkers in their labeling in last 26 months, compared with 46% in Hungary. However, the timelines used by the Hungarian authorities to update SmPCs according to FDA drug labels are hard to predict.

In this study, FDA listed drugs (*n* = 264) with pharmacogenomic biomarkers in drug labeling were compared with drugs in the Hungarian National Institute of Pharmacy and Nutrition database with potential pharmacogenomic information in their SmPCs. Some active ingredients in Hungarian SmPCs may exist with pharmacogenomic information, although not mentioned by the FDA. These drugs remained hidden in our study.

According to a previous study, pharmacogenetic information is included in patient-targeted sections for a minority of drug labels [24]. Our research focused on drug labels’ doctor targeted section, but rather superficial content of patient information leaflet was ignored.

Original active agents were investigated in the study. Differences between original and generic drug’s label were neglected.

This study was performed in support for regulatory decisions. In order to minimize the drug-associated risks in the general Hungarian population and reduce uncertainties about application of PGx biomarkers for medical practitioners.

## Supplementary information

Supplementary Table 1
