# Supplementary material for: Pharmacogenomic biomarker information differences between drug labels in the United States and Hungary: implementation from medical practitioner view
Source: Pharmacogenomics J. 2019 Dec 2;20(3):380–7. doi: 10.1038/s41397-019-0123-z (PMC7253355; doi:10.1038/s41397-019-0123-z)
Supplement: Supplementary file 1 — Supplementary Table 1 [file 41397_2019_123_MOESM1_ESM.docx]

Supplementary Table 1. Drugs not available in Hungary (n=69) from the 264 FDA listed drugs with pharmacogenomic biomarkers in drug labeling

| Ado-Trastuzumab Emtansine  Amoxapine  Amphetamine  Arformoterol  Aripiprazole Lauroxil  Ascorbic Acid  Atezolizumab  Belinostat  Boceprevir  Carisoprodol  Cevimeline  Chloroprocaine  Chlorpropamide  Dapsone  Denileukin Diftitox  Desflurane  Desipramine  Desvenlafaxine  Deutetrabenazine  Doxepin  Dronabinol  Duvelisib  Elagolix | Emapalumab  Enasidenib  Enflurane  Eteplirsen  Flibanserin  Fosphenytoin  Gilteritinib  Glipizide  Glyburide  Hydralazine  Hydroxychloroquine  Iloperidone  Isosorbide Dinitrate  Ivacaftor  Ivosidenib  Ixabepilone  Larotrectinib  Lofexidine  Mafenide  Meclizine  Modafinil  Nalidixic Acid  Nefazodone | Nortriptyline  Omacetaxine  Pegloticase  Perphenazine  Pimozide  Primaquine  Probenecid  Procainamide  Protriptyline  Simeprevir  Sodium Nitrite  Succimer  Succinylcholine  Sulfisoxazole  Tafenoquine  Telaprevir  Thioguanine  Thioridazine  Tolazamide  Tolbutamide  Tolterodine  Trimipramine  Valbenazine |
| --- | --- | --- |

The table represents the status of 2019 July;
